# Compact or spread? A quantitative spatial model of urban areas in Europe since 1990

**DOI:** 10.1371/journal.pone.0192326

**Published:** 2018-02-28

**Authors:** Manuel Wolff, Dagmar Haase, Annegret Haase

**Affiliations:** 1 Department of Geography, Lab for Landscape Ecology, Humboldt Universität zu Berlin, Berlin, Germany; 2 Department of Urban and Environmental Sociology, Helmholtz Centre for Environmental Research – UFZ, Leipzig, Germany; 3 Department of Computational Landscape Ecology, Helmholtz Centre for Environmental Research – UFZ, Leipzig, Germany; Public Library of Science, UNITED KINGDOM

## Abstract

Changes in urban residential density represent an important issue in terms of land consumption, the conservation of ecosystems, air quality and related human health problems, as well as the consequential challenges for urban and regional planning. It is the decline of residential densities, in particular, that has often been used as the very definition of sprawl, describing a phenomenon that has been extensively studied in the United States and in Western Europe. Whilst these studies provide valuable insights into urbanization processes, only a handful of them have reflected the uneven dynamics of simultaneous urban growth and shrinkage, using residential density changes as a key indicator to uncover the underlying dynamics. This paper introduces a contrasting analysis of recent developments in both de- and re-concentration, defined as decreasing or increasing residential densities, respectively. Using a large sample of European cities, it detects differences in density changes between successional population growth/decline. The paper shows that dedensification, found in some large cities globally, is not a universal phenomenon in growing urban areas; neither the increasing disproportion between a declining demand for and an increasing supply of residential areas nor actual concentration processes in cities were found. Thus, the paper provides a new, very detailed perspective on (de)densification in both shrinking and growing cities and how they specifically contribute to current land take in Europe.

## 1. Introduction

The concepts and discussions about the direction and dynamics of urbanization, such as growth or shrinkage, have one issue in common: They deal with the relationship between built-up areas in urban areas and the population concentrated in them. Or, in other words: about urban population density. This paper aims to improve the understanding of residential density changes, called density changes in the following, in urban areas by comparing European countries during the very dynamic, recent period between 1990 and 2010.

Ongoing land consumption and sprawl at the edge of European urban areas contradicts the preferred planning normative of the ‘compact city’ [1,2] and, instead, leads to ‘donut cities’ as a consequence of a hollowed-out core and a growing hinterland [3,4]. Studies have shown that sprawl is strongly linked to either increasing per capita land consumption, driven either by income growth of households and of the national GDP [5] or by the availability and price of land [6]. The comparatively low costs of car-based mobility mean that those urban areas that are growing faster and expanding are experiencing an exodus of people and jobs to their hinterland [7,8]. The effects are decreasing densities that are most commonly described as dedensification or ‘deconcentration’. Contrary to this sprawling development, a new, return flow of small and high-income households targeting (inner) cities is driving a novel (re)growth and (re)densification of the urban area, overcoming patterns such as donuts or urban perforation [9,10,11,12], which, in turn, means new ‘concentration’ within the city borders.

Both deconcentration and (re-)concentration belong to urbanization and the respective population density concentrated in the urban built-up area. These changes of density are an important issue in urban and land-use research [13,14,15] and one major aspect of the global sustainability discussion [16], because it provides evidence on how much land and resources dedicated to this land are taken by urban dwellers, relative to their number. This can be linked to human quality of life and the ecological footprint of this quality of life [17], because urban areas may use more or less primary resources or be more or less efficient in resource use when maintaining a defined threshold of quality of life for their residents [18].

Therefore, assessing residential density changes is essential for evaluating land consumption, the conservation of natural capital and ecosystems, air quality and related health problems in cities, and the corresponding challenges for urban and regional planning [19]. In this respect, residential density is a simple but powerful indicator for urban growth/decline [20], the patterns that these trends reveal for the city, and how they change over time [21,22,23,24,25].

To date, a range of studies has provided valuable insights into the expression of various indices, to describe concentration and deconcentration in European cities; these include continuity and centrality, clustering and nuclearity, concentration and density [26,27]. Thereby, residential density is regarded as a powerful indicator for urbanization because it reflects the relationship between the supply of built-up area and the demand for it by a certain number of people [28]. Basically, there are three categories of studies that have dealt with residential density. In the first category, density is used to define and delineate territories, e.g., differentiating between urban and rural areas [29], defining specific areas [30], or distinguishing between different degrees of the urban character [31]. The second category, uses residential density to describe and compare urban systems, such as those found in Europe and the US, from a historical [32], spatial [33,34,35], or socio-demographic perspective [36]. In the third category, density is be used to describe processes and phenomena of urban evolution [37]. Deconcentration and dedensification can be parallelized—at least to a certain extent—with what we know as ‘urban sprawl’ [7], a phenomenon that has been extensively studied in the US [38, 39,40,41,42,43] but also globally [6,14,44,45]. In Europe, in addition to sprawl and in accordance with what happened in the US from the 1960s to 1980s, the well-known concept of the ‘in-between city’ (Zwischenstadt)–something in between ‘the urban’ and ‘the rural’–has also been discussed [46,47]. Sprawl was studied in Europe basically by focusing on particular regions or even cities [48,49,50,51,52], but recent studies have started to pay more attention to a cross-country comparative perspective that covers a range of cities [5,20,53,54,55,56,57,58].

Thereby, density was used to study spatial patterns of European cities, using geo-spatial indices and landscape metrics [53,59], in order to identify factors that influence the multidimensional character of sprawl [5] or to explore the relationship between urban sprawl and a set of variables by testing urban economic theories [54]. Similar to a comprehensive study on understanding urban land-use changes in European countries, conducted by Siedentop and Fina [55], Kasanko et al. [20] understand urban density as a measure of land-use intensity. The authors used an indicator framework that led from basic land use indicators to population density measurements and, finally, to a combined analysis of population densities and land-use intensity. There is a long tradition [60] of using density as an indicator in order to measure the compactness and intra-urban population distribution using a population density gradient starting at the city centre and following a negative exponential [61,62], quadric exponential [63] or power function [64]. However, these models mostly aim at testing the fit of the distribution and provide explanations of density changes over space rather than over time (e.g. [65]). Yet other studies have specifically focussed on typologies of urban development in Europe. Based on the prevailing characteristics of land-use changes at the regional level, the LUPA project defines three main types of cities (with slow, rapid, and very rapid growth) that are, amongst other features, also characterized by a ratio of built-up area to population [66,67]. To our knowledge, a typology developed by the Netherlands Environmental Assessment Agency is the only study that specifically combines density changes with (rapid) population growth/decline, in order to identify and assess urbanisation patterns [68,69].

**Fig 1:**
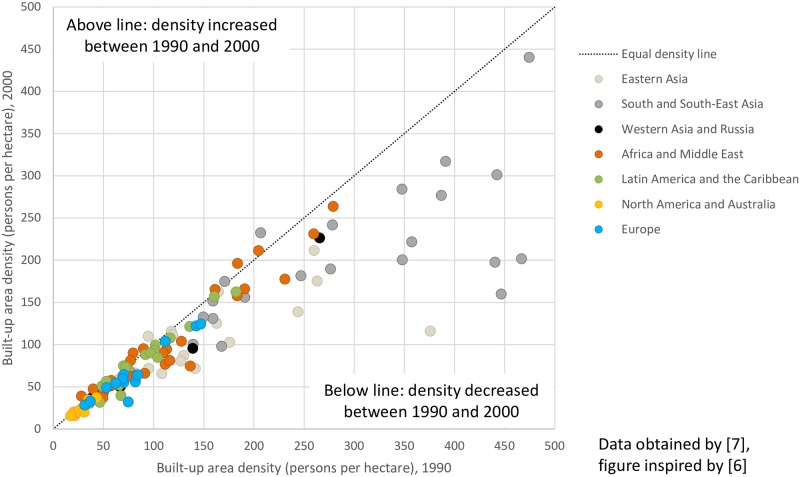
Development of average density in built-up areas, in a global sample of 120 cities, 1990–2000 [6].

An impressive picture of density changes for major cities across the globe was drawn by Angel et al. who applied models that explain variations in density [6,7,70]. At the core of their model, the authors plot the density of built-up area for one time period against the density of another period, as shown in Fig 1 [6]. They demonstrated that population growth leads to deconcentration and dedensification in cities across all continents, including Europe, as urban land area expands faster than the population grows [7,17]. Angel et al. [6] emphasized that urban densities generally decreased and will continue to do so in the future. Although this model makes it possible to draw conclusions only about quantitative aspects of density changes, it has, compared to other concepts, three basic advantages:

- Although Angel et al. [7] focused on urban expansion or sprawl, the process-oriented characteristic of the model also allows the study of concentration processes, thus providing the opportunity to test hypotheses about driving forces with tools such as independent t-tests.
- At its core, the model uses changes of built-up area density, which represents “the most robust measure of urban expansion” and reveals whether a city is developing in a more compact way and with less sprawl than other cities [70]. Although density changes fall short in describing sprawl in all its dimensions, they still represent a meaningful and illustrative reference for, e.g., policy consultations. Density changes provide a relatively comprehensive characterization of urbanization processes that can be further elaborated and analysed systematically.
- The authors graphically illustrate the density changes with a diagonal line that shows where the density in two periods is exactly equal. This also allows the creation of subsamples with respect to city size, to location in developed and developing world, or to population growth or shrinkage. Furthermore, it easily reflects the amount of density change, thus enabling conclusions about the intensity of the process to be drawn [20,55].

However, only a few studies use density (change) as a key indicator to characterize urbanization processes [6,9] and, despite the large variety of the studies mentioned above, our knowledge about urban (de)densification is limited for the following reasons:

1. First, many studies focus on declining density and discuss urban sprawl or scattered urbanization [53,54,55,71]. Thus, they poorly reflect the uneven dynamics of simultaneous population growth and shrinkage in cities. Europe, in particular, is the continent where most shrinking cities can be found [72], including urban land abandonment and land use perforation as a consequence of post-industrial land use changes and demographic ageing [10,73,74]. Besides shrinkage, a considerable number of cities in Europe have experienced recent reconcentration processes [11,12]. Thus, different density pathways have to be expected [9,20].
2. Second, there is still a gap between observation and theory. Although theoretical considerations are predominantly based on economic models of urban spatial structure [75,76,77,78,79] a theory that explains density changes of urban areas is not fully developed [7]. Several authors emphasize that the basic drivers, such as physical, demographic, economic, technological, and political factors, only explain density changes to a certain extent [6,17,41]. In particular, the role of population growth as an explanatory factor is not clear [17,54]. Thus, further investigation of the relative contribution of density change (mean population and residential area) is required in order to fully understand the specific conditions under which European cities are developing [7,54].
3. Third, the explanatory power of the studies discussed above is restricted to large cities, thus providing a rather selective picture of the urban hierarchy [17] and underestimating the importance of small and medium-sized urban areas. This is, on the one hand, due to limited data availability, especially for small-scale and comparable population data covering different points in time [5,80]. On the other hand, all concepts of urbanization are somehow confronted with a heterogeneous set of definitions of what, across Europe, an urban area is [80]. However, a large segment of the urban population in Europe lives in small or medium-sized urban areas, which play an important role in terms of economy, service and infrastructure provision, or the well-being not only of their own inhabitants but also of the rural populations surrounding them [81]. Thus, also considering small cities is important for analysing the balanced spatial development and cohesion of the European territory, and also in terms of their different expressions of density changes [5,7,74].

Against this background, this paper presents an analysis of recent urbanization in Europe, with a focus on both deconcentration and concentration with respect to population growth or decline, taking into account small and large urban areas. Due to its advantages, we use the density change model developed by Angel as the starting point of our analysis. This will then be extended by drawing attention to differences between growing and shrinking urban areas in Europe that have experienced physical de- and (re)concentration during the very dynamic period between 1990 and 2010 [53,55,74]. Europe has been chosen because it displays urban growth and shrinkage that is combined with the world’s strongest demographic aging tendencies. These processes justify the relevance of our current analysis that were, however, hardly reflected in the model of Angel et al. [6,7]. This allows us to evaluate the relative contribution of the factors contributing to density change and to discuss the approach of Angel et al. An extended discussion of density change under conditions of growth and is also crucial for supporting policy makers who decide on infrastructure adaptation or modes of governance. Set against this background, the paper does not aim to explain sprawl but rather to improve the understanding of changing residential density that depends on growth and shrinkage by answering the following questions:

- Are residential densities in urban areas across Europe generally declining, as the model of Angel et al. [6] suggests?
- To what extent do we find differences in density change between growing and shrinking cities, as well as between small and large ones? And,
- Which driving factors might explain the observed trends?

## 2. Materials and methods

Residential density, as a measure of land-use intensity, is not an easily interpretable concept [42]. For measuring densities the paper uses the term ‘urban areas’ in line with other authors [20], in order to reflect a conurbanisation covering several municipalities, on the one hand, and to avoid confusion about the different terms used in different countries, e.g., cities, towns, agglomerations etc., on the other. We built on the model of Angel [6,7] to calculate density changes in urban areas but extended it in two ways to address the prevailing shortcomings of other concepts. First, we developed an approach for delineating urban areas and, second, we extended the measurement concept in order to reflect on the relative contribution of the components of density changes. The paper uses ‘residential density’, calculated by total population per residential area. This has clear advantages compared to the concept of net-density [82,83], allows comparisons to other studies [20], allows to detect reuse [84] and focuses on the area for residential housing a city requires for inhabit their residential population whereas taking into account further land uses such as industrial areas would to some extend blurs the conclusions on the relation between residents and their available housing.

In order to delineate cities, Angel et al. [6] focused on (morphological) urban areas, which are used when studying land-use patterns and for analysing the planning of infrastructure systems. This morphological approach increases the comparability between municipalities which vary in size and shape by merging administrative units, based on a continuous built-up area and summing up the population number of the merged administrative units [6,7,55]. In Europe, essentially two cross-country databases are available: the Morphological Urban Areas (MUA), which was created in 2007 and updated in 2011 by IGEAT, and the Urban Morphological Zones (UMZ), created by the European Environmental Agency in 2002. The MUAs are defined for the reference year 2001 basically by selecting the most densely populated municipalities (LAU2) with more than 650 inhabitants per km^2^ and with a minimum population exceeding 20,000 inhabitants [85]. The database comprises 1988 units in 29 countries (EU27, Norway and Switzerland). The UMZs are composed of continuously built-up areas which is derived from Corine Land Cover [86,87], containing "urban fabric" (continuous or discontinuous), "industrial commercial units", "green urban areas", certain forest spaces, and port areas; airports, sports and leisure facilities, and road and rail networks (for more details about the construction rules, see [87,88]). This results in approximately 4300 units with a minimum population of 10,000 inhabitants covering 29 countries (EU28 and Liechtenstein). However, both databases are of limited use. The population data provided for MUAs show some gaps and some results for the smallest units should be interpreted with caution. Whereas MUAs focus on large cities and tend to over- or underestimate the actual cover of built-up area (see S1 Fig), UMZ units lack time series of population data and are not applied to administrative units. However, this aspect is necessary in order to compare the units with Angel’s approach and also because policies at the administrative level may also influence the evolution of cities [66].

**Fig 2:**
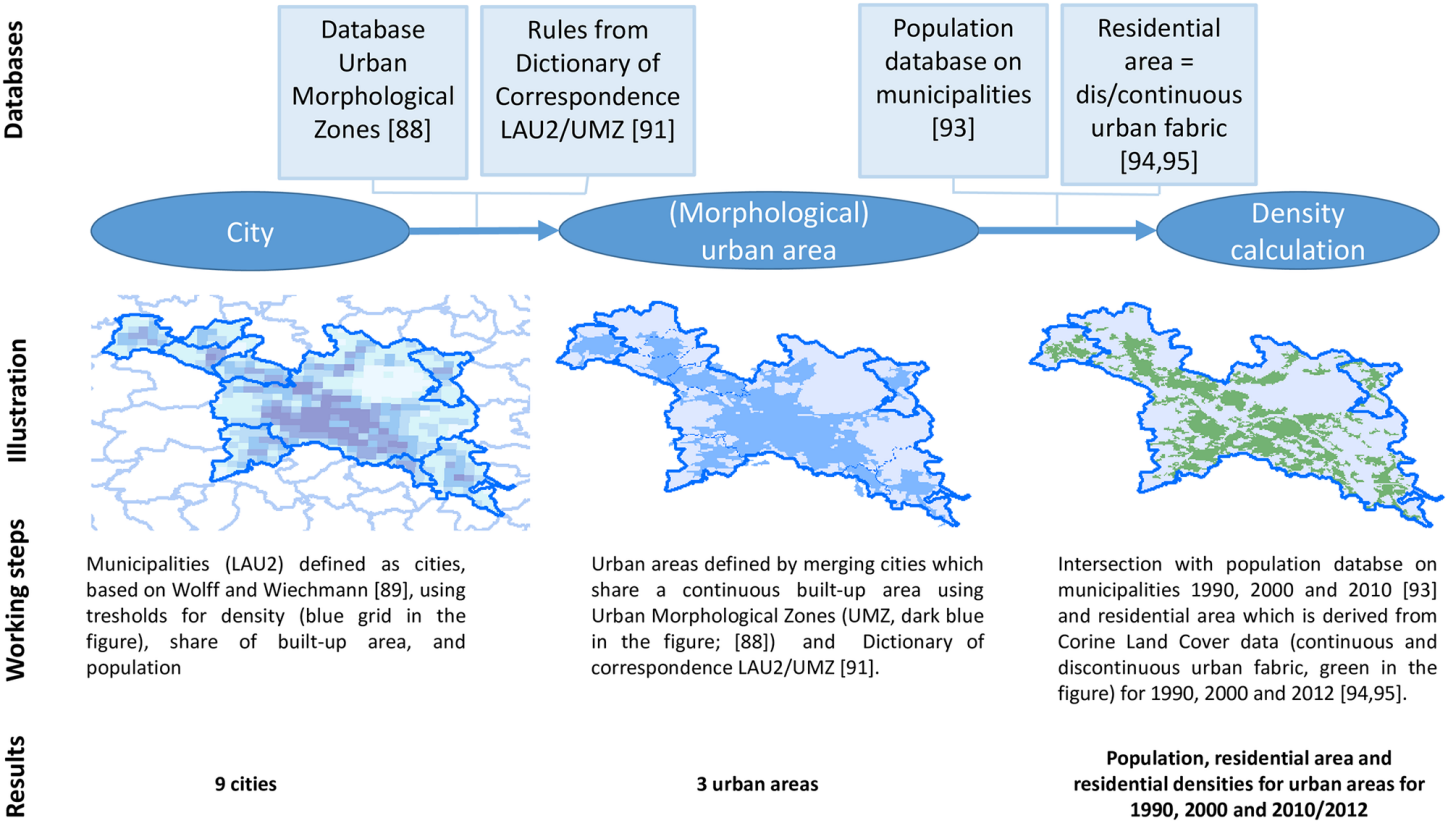
Workflow of the approach.

Thus, we ***developed an approach for delineating urban areas*** that covers small to large urban areas. Thereby, we used the strengths of the UMZ database and combined it with an approach developed by Wolff and Wiechmann [89], which defined municipalities (LAU2) as cities according to OECD-EC guidelines using a minimum population, density and share of built-up area [31,90]. Fig 2 shows that morphological units are delineated by merging the defined cities with their adjacent non-urban municipalities, based on the rules developed for the Dictionary of Correspondence LAU2/UMZ [88,91]. This approach results in 5,692 (morphological) urban areas in 35 European countries. S1 Table in the supporting information provides some basic indicators for these countries. A comparison of the median values of population size and surface of residential area (urban fabric) reveals that the units are comparable among European countries (S2 Fig). However, it has to be noted that the low densities in the Scandinavian countries, Bulgaria, and Serbia are basically due to the relatively large units. A selective picture of large urban cities is only obvious for the United Kingdom and Lithuania and needs to be considered when interpreting the results. However, as the number of urban areas is low, the overall picture of the results is not distorted.

***Densities were measured*** as the ratio of the total urban population and the total residential area [37,41,42,43,92]. Angel et al. [6,7] used the total built-up area but in line with Kasanko [20] we used the residential area in order to better mirror population concentrated in their residential areas. In contrast to net or urban density, the values of residential densities are usually larger as the same population size is referred to a smaller reference area (residential instead of total built-up area, see also [7] p. 39). In order to exclude fluctuations, a change in the resident population and built-up area exceeding 1% was registered. Population numbers were derived from a database of population figures for municipalities for 1990, 2000 and 2010 [93] that had been linked to the urban areas (Fig 2). Residential area was obtained from Corine Land Cover data for 1990, 2000 and 2012: we used continuous and discontinuous urban fabric as the residential land uses [94,95]. In line with other studies [20,54,55,59], Corine was chosen because it is, currently, the most recent source providing residential land-use data also for very small areas, whereas UMZs provide data for the entire urban built-up area, also including industrial areas, and only for units of more than 10,000 inhabitants. It should be noted, however, that urban areas smaller than 25 hectares especially in rural areas are not covered and thus urban area is under-represented compared to the official statistics [67,96,97].

**Fig 3:**
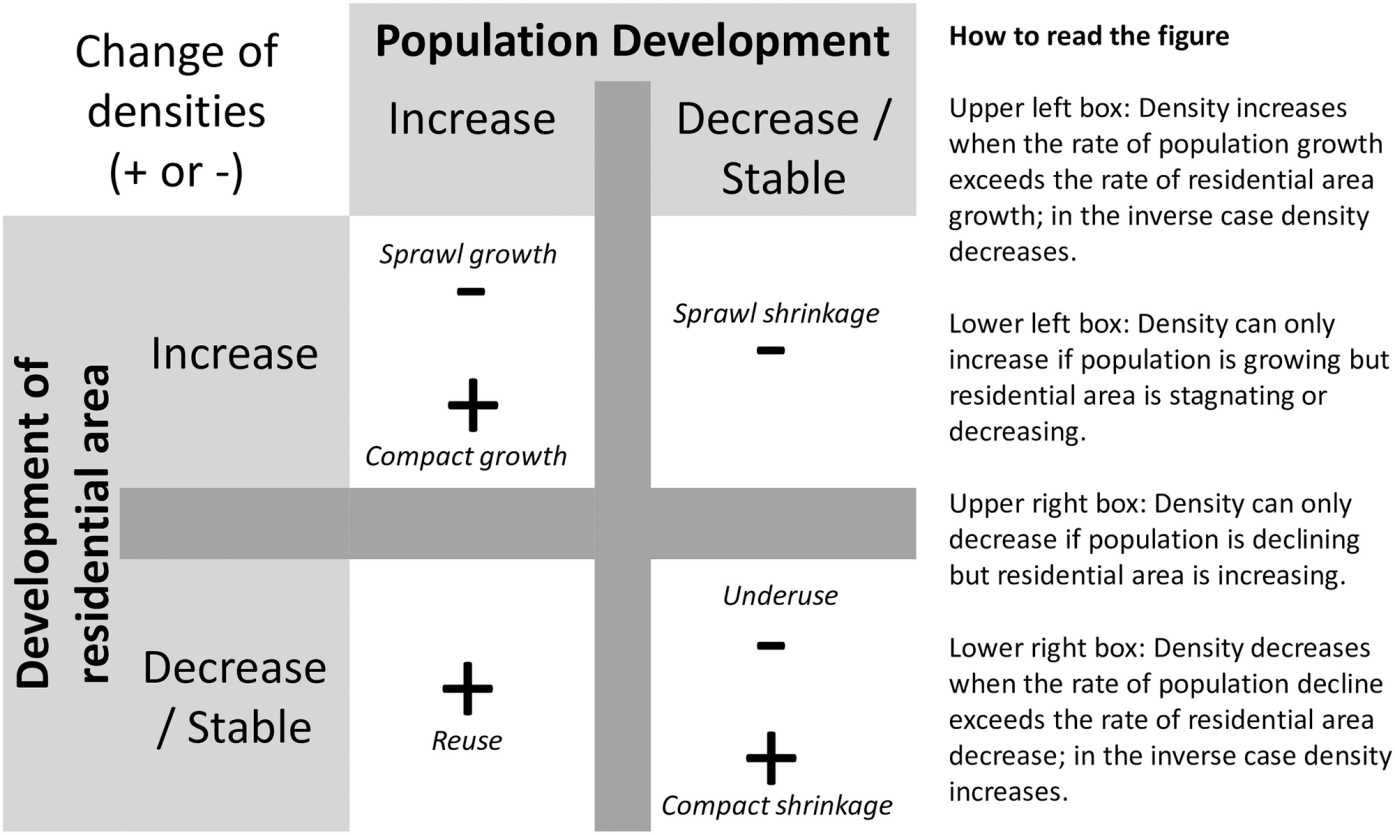
Operationalisation of population density changes under different outcomes of urban population development and built-up areas [9].

Due to the unambiguous relationship between growth of population and residential area [17,80], we applied an ***extended measurement concept*** for density changes, by investigating the relative influence of changes in the population and the residential area, in order to draw conclusions about the underlying driving forces (Fig 3, for details see [9]; similar considerations also in [20]). Changes in residential area reflect the changing physical extent of the urban area and allow the discussion of phenomena such as sprawl, whereas population allows one to set these cities into the context of the debate on growing and shrinking urban areas.

As Fig 3 shows, both a change in population and/or residential areas can result in changes in density. For declining population numbers in urban areas, densities most probably decrease, leading to an oversupply and underuse of urban land and the associated demolition of buildings [98]. However, if the rate of the demolished area exceeds the rate of population loss, due to, e.g., the demolition of large prefabricated buildings, densities can also increase (compact shrinkage). Furthermore, some studies even show an extension of residential areas (e.g., newly built houses) although population numbers have fallen, leading to a paradox situation with increasing densities—in other words: sprawl—under conditions of shrinkage [4,80].

Increasing densities are usually associated with compact population growth, infill, and new residential areas, which may encourage savings in operational costs for public services, such as schools and water/wastewater utilities [50]. In particular, a constant physical expansion of a city, with newly built housing, can even lead to decreasing densities, which is a common characteristic of many cities worldwide, leading to sprawl-like growth [7,98]. The consequences are longer commuting distances and less access to public transport, resulting in a greater use of private cars [99,100]. In contrast, in urban areas experiencing new growth, in particular, densities can increase without an expansion of the built-up area [101,102], even in the context of ongoing demolition of some decayed buildings, due to reuse and renovation of the existing housing stock that was previously vacant and is now absorbing the increasing demand.

In accordance with other studies, we used population as a common indicator for urban dynamics, in order to define shrinking urban areas by a population loss of more than 0.15% p.a. (and vice versa for growing cities) [74,103,104], and to distinguishing between large and small urban areas, using a threshold of 100,000 inhabitants, in order to allow comparisons with studies by Angel and others [6,7,81]. Thereby, we applied t-tests that investigated differences between growing and shrinking as well as large and small urban areas in terms of their density changes.

## 3. Results: Density changes in Europe

### 3.1 Comparing changes in population, built-up areas, and density

In line with other studies, urban growth is prevalent in Europe; approximately 70% of all urban areas experienced population growth between 1990 and 2010 (S2 Table; [73]). Although one third of all urban areas (29%) experienced population loss over the same period, we only observed a significant reduction in residential area for just 9% of all urban areas. Moreover, although the residential area of 76% of all urban areas increased, density decreased in almost 40% of urban areas, therefore reflecting deconcentration. Thus, we need to focus on the extent to which deconcentration and dedensification are universal for growing urban areas. We applied the model of Angel et al. [6,7,70] for the periods 1990–2000 and 2000–2010 and differentiated between growth and shrinkage, as well as between small and medium-sized (<100,000 inhabitants) and large (>100,000 inhabitants) urban areas, because Angel analysed larger ones.

**Fig 4:**
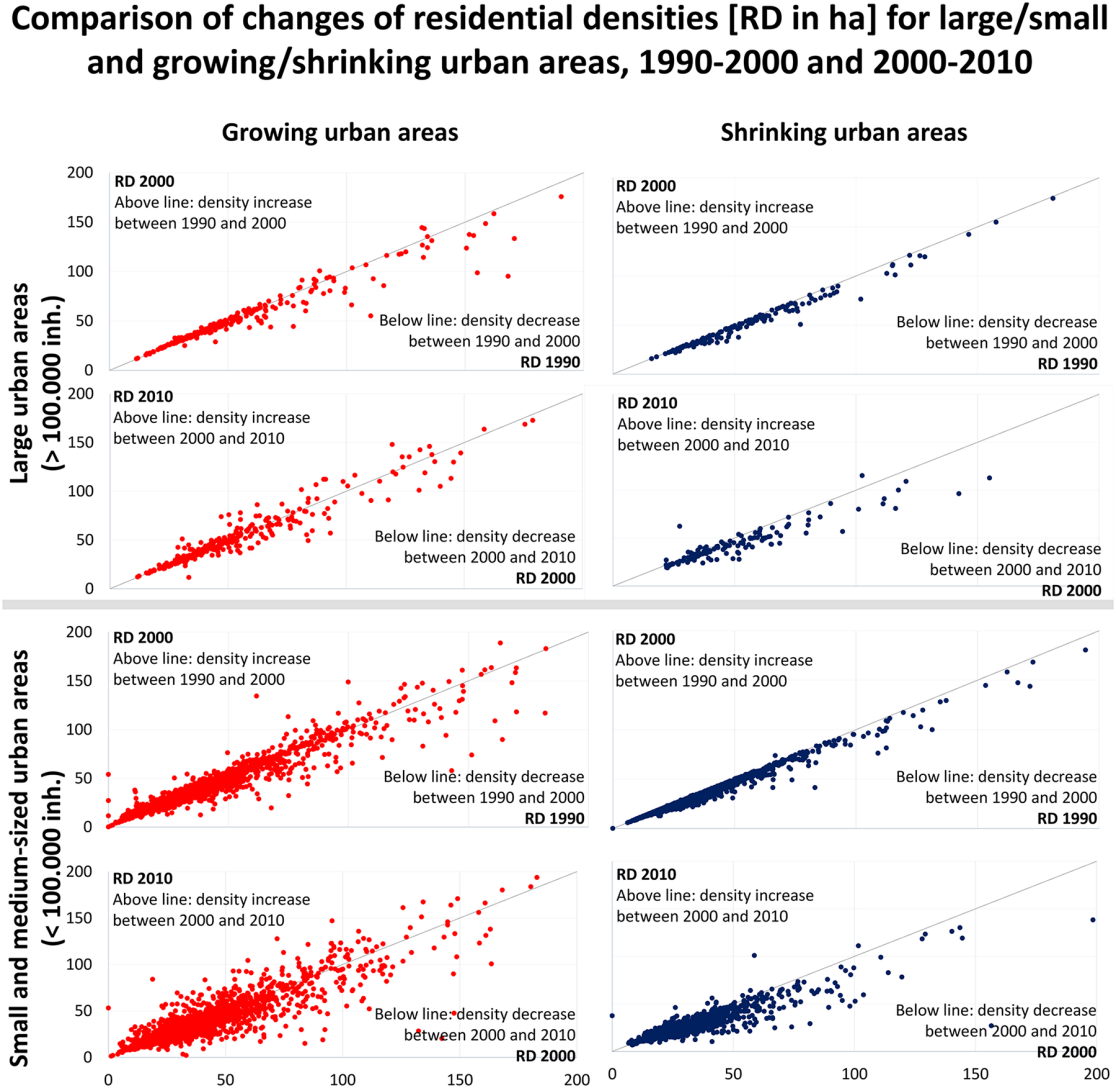
Density changes 1990–2010 for large and small urban areas.

In the decade from 1990 to 2000, growing urban areas experienced the most rapid density changes (Fig 4), especially in very dense urban areas (>100 inhabitants/hectare). In line with Angel et al. [6,7], we found sprawl and dedensification among growing European cities. During this period, however, two other processes emerged: On the one hand, all shrinking urban areas contributed to this dedensification process. On the other, a substantial number of growing cities also exhibited increasing densities with, however, very low rates of density change (Table 1).

**Table 1:** Frequencies, average changes for four types of density changes and f-tests for differences between growing and shrinking urban areas.

	All Urban areas		Large urban areas		Small and medium-sized urban areas	
	1990–2000	2000–2010	1990–2000	2000–2010	1990–2000	2000–2010
**Absolute frequencies** (Frequencies can vary because a threshold of +/- 0.15% was applied for changes both in densities and population.)						
growth/dedensification	1,028	1,766	124	149	904	1,617
growth/densification	2,704	1,642	120	137	2,584	1,505
shrinkage/dedensification	1,568	1,162	156	74	1,412	1,088
shrinkage/densification	6	767	0	58	6	709
**Average values of density changes [%]**						
growth/dedensification	-8.6	-41.1	-8.4	-37.9	-8.6	-39.8
growth/densification	8.1	113.5	3.5	92.7	8.3	107.8
shrinkage/dedensification	-6.8	-40.6	-7.0	-40.3	-6.8	-38.5
shrinkage/densification	3.7	78.2		75.6	3.7	68.7
**F-Test for differences of density changes between growing and shrinking urban areas**						
*F-Value*	**0.12**	**6.5**	**5.69**	**2.42**	**0.05**	**9.58**
*Significance*	0.73	0.01	0.02	0.12	0.82	0.00

After the year 2000, we observe three parallel and accelerating trends: dedensification in growing as well as shrinking urban areas, and densification in growing urban areas.

The trend of decreasing densities was reinforced because the number of ***growing and dedensifying urban areas***, as well as their density change rates, significantly increased (numbers increased by 66%; change rates by -33% points; Table 1) in both small and medium-sized as well as large urban areas. This is in line with other studies, which underline that urban sprawl is most likely to continue, resulting in less compactness [6,19,20]. Although population rates are generally increasing more slowly in larger than in smaller dedensifying growing urban areas [20], there is very little difference between small and large or between dense and less dense urban areas, in terms of density change rates, which contradicts the findings of Angel et al. [7] to a certain extent.

Apart from these findings fast, ***shrinking urban areas*** contribute to this dedensification process in both periods with rates exceeding those of growing urban areas. This can be observed especially among less dense urban areas (<100 inhabitants per hectare), but the denser a shrinking urban area is, the more likely it is to follow a decreasing density change (Fig 4). This dedensification process in shrinking urban areas shows similar values, on average, to growing ones, although they experience a gain in the population at a faster rate than shrinking urban areas experience a fall in the population (Table 1). Although the number of shrinking cities increased (from 29 to 36% between the two periods; see S2 Table), the number of dedensifying shrinking urban areas decreased after 2000, because of a new phenomenon. A substantial number of urban areas experienced a very rapid increase in densities, despite the fact that population numbers were still falling significantly overall. As a consequence, we observed a trend towards more compactness in the context of shrinkage in both large as well as small and medium-sized urban areas.

The third trend mirrors a concentration, or the ***densification of growing urban areas***, which was already observed before 2000. However, the average change in density rates of these urban areas increased significantly between 2000 and 2010 and even exceeded those of dedensifying growing urban areas (Table 1). Moreover, after 2000, a significantly increasing polarization between large and small urban areas is obvious. Whereas the number of growing, densifying urban areas with less than 100,000 inhabitants decreased, their number in more populated areas substantially increased. The f-test revealed that differences in density change rates between growing and declining large urban areas decreased and even reached a non-significant level, which actually points to a reinforced densification process (Table 1). We can say, with 95 percent confidence (significance ≤ 0.05), that, on average, density changes differ by 9.58% between growing and shrinking urban areas between 2000 and 2010. In contrast, the differences between growing and shrinking small urban areas increased significantly as the result of simultaneous population growth and decline and associated densification and dedensification processes in these small and medium-sized urban areas. This is in line with previous studies that demonstrated a redensification of several European cities [20].

### 3.2 Typology of density change

Whereas the previous chapter bridges the results of Angel et al. [6,7,70] with an in-depth continental sample and identifies three basic density change trends, this chapter investigates how successional shrinkage and (re)growth have an impact on density change. Therefore, we apply the extended measurement concept and shed light on the underlying dynamics of density changes by focusing on the contribution of changes in the population and in residential areas.

Dedensification and deconcentration are basically driven by the disproportionate development of physical expansion and population development that involve two phenomena: ***extended urban sprawl and sprawl without growth***. First, the phenomenon of urban sprawl or deconcentration, which was already described by Angel et al. [6], counts for more than one third of urban areas; their corresponding share increased from 34% prior to the year 2000 up to 37% between the years 2000 and 2010. Table 2 reveals that, in an increasing number of European urban areas, including small and medium-sized as well as larger ones, residential areas increased faster, in relative terms, than population numbers (leading to sprawl). The share increased markedly in those countries with existing extensive urban sprawl, such as Portugal, Greece and France, or remained high in the Netherlands, Spain or Italy. Second, the disproportion between an expanding built-up area but declining population numbers led to the paradox of shrinking urban areas contributing to the dedensification process (sprawl shrinkage), which was not covered in Angel’s model [6,7]. The data presented in Table 2 show that every fifth urban area follows this sprawl without growth and that the percentage even increased (from 22% to 27%), especially among small and medium-sized urban areas, and fell among larger ones. The percentage of these urban areas with ‘sprawl-without-growth’ dominates in post-socialist countries, e.g., the Baltic States, Poland, the Czech Republic, and increased not only in Serbia, Slovakia, and Croatia but also in Germany. In Western Europe, however, the percentage of urban areas of this type was high before the year 2000, e.g., in Italy and France.

**Table 2:** Frequencies of urban areas with density changes (+/- in heuristic) in different constellations of changes to the population and residential areas.

	Frequencies *(relative)*		Frequencies *(absolute)*		Heuristic of density changes			
							Population development	
							*Increase*	*Decrease/Stable*
**1990 to 2000**	34.3%	22.3%	1,047	680	**Development of residential area**	*Increase*	**+** Sprawl growth	**-** Sprawl shrinkage
	40.5%		1,237				**-** Compact growth	
	2.0%	0.2%	60	7		*Decrease /Stable*	**+** Reuse	**-** Underuse
		0.7%		22				**+** Compact Shrinkage
**2000 to2010**	37.2%	27.0%	1,919	1,395	**Development of residential area**	*Increase*	**+** Sprawl growth	**-** Sprawl shrinkage
	17.7%		916				**-** Compact growth	
	9.2%	4.5%	476	231		*Decrease /Stable*	**+** Reuse	**-** Underuse
		4.4%		225				**+** Compact Shrinkage

Whereas, between 1990 and 2000, the residential area was hardly reduced in any urban area but, instead, expanded (S2 Table), in more than 4% of all urban areas we observed a ‘physical adaptation’ in terms of decay and ***demolition of buildings*, *leading to decreasing densities*** (underuse, 4.5%). The decline in densities caused by these demolition activities is another trend that is not covered by the model of Angel et al. [6,7,70] but it shows that population loss occurs faster than the adaptation of the physical infrastructure. This trend is observed in Bulgaria and Romania but also in peripheral regions of the Baltic States, Austria, or Slovakia. Furthermore, in terms of large-scale demolition programs, such as in (Eastern) Germany, the rate of demolition can even exceed the rate of population loss, leading to the paradox of increasing densities in shrinking urban areas, especially in those urban areas that had already lost a substantial number of residents before demolition started [9]. This type of ‘right-sizing’ or compact shrinkage accounts for 4.4% of all urban areas and is also associated with the reduction of residential areas for infrastructure, commercial, or residential purposes through conversion to other forms of land use such as parks [105], which—in absolute numbers—is particularly visible in Germany. High shares can also be found in Bulgaria and Romania, as well as in Serbia and Slovakia.

**Fig 5:**
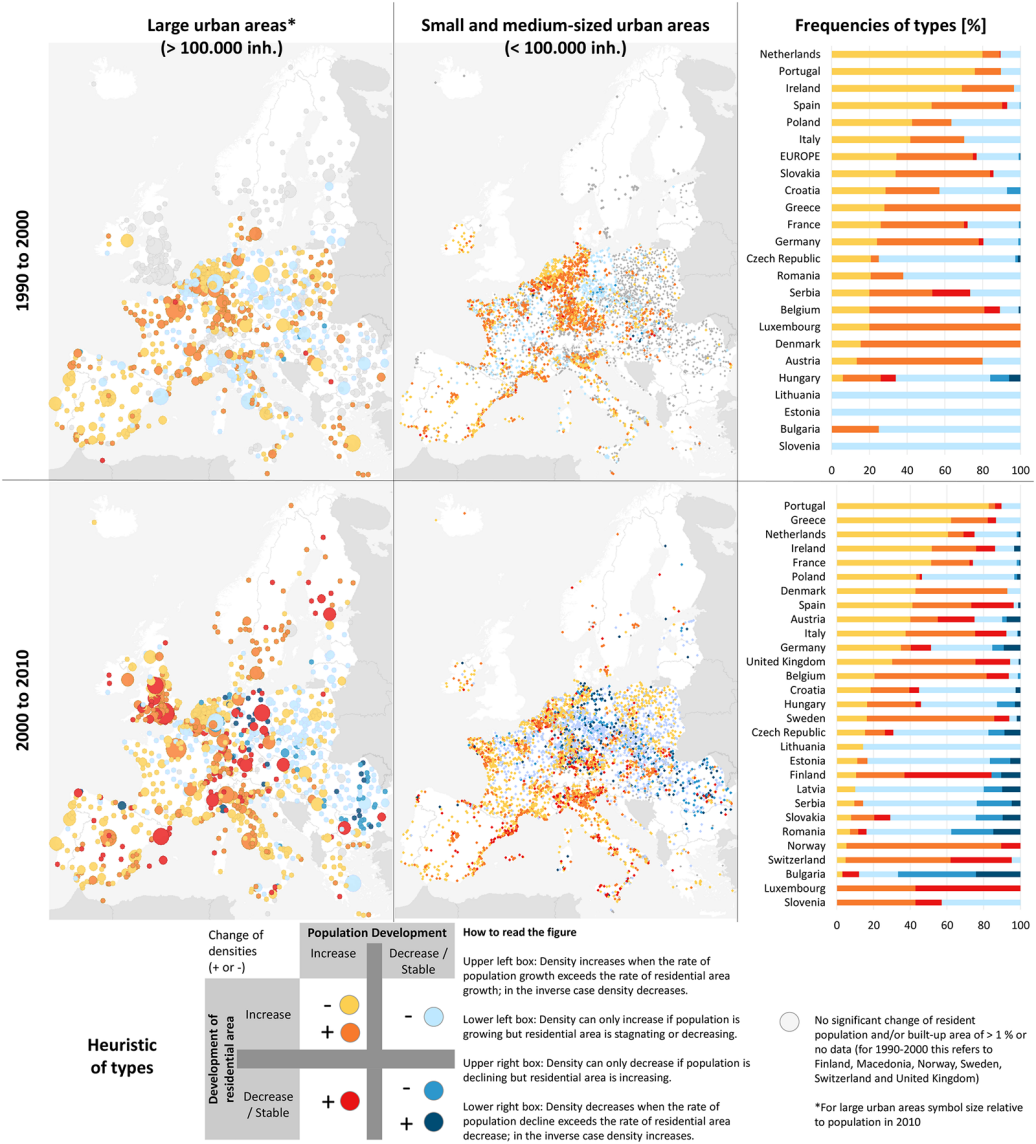
Spatial distribution and frequencies of density change patterns.

Apart from this, ***densification processes*** are a characteristic of growing cities. A large number of urban areas reflect rising residential densities due to a rapid increase in the total population, indicating the emergence of ‘compact growth’ with a lower per capita flat area, compared to sprawling urban areas. This trend can be observed particularly in Norway and Sweden, as well as in Belgium, the UK, Switzerland or Northern Italy. For Europe in general, however, the percentage of urban areas displaying this trend decreased substantially from around 40% to 18%–especially in small urban areas—e.g., in France, Greece, Germany, Austria, and Slovakia, in favour of sprawl (or slower population growth). In contrast, a new phenomenon can be observed that characterizes cities with renewed growth, in particular [9]: density increase in growing urban areas without an expansion of residential areas. A stable or even slightly decreasing amount of residential areas can be measured as a consequence of the refurbishment of large parts of the housing stock that used to be vacant (reuse). Overall, we find this kind of densification in a large number of European urban areas after the year 2000 (10%); it is a clear sign of regrowth, which has been discussed as reurbanisation or urban resurgence in the scholarly literature [12,106,107,108,109]. Fig 5 shows that renewed growth in urban areas does not represent a major trend in any country or shows characteristic spatial concentration in regions. However, it is important to realize that this is an emerging phenomenon of increasing importance for Europe [73] and that it predominantly characterizes large urban areas, such as those in Germany, the UK, or Spain, and can even be found in post-socialist European countries that used to belong to the ‘pole of shrinkage’ in the 1990s and 2000s.

## 4. Discussion

To discuss our results, we return to our three initial research questions.

### Are residential densities in urban areas across Europe generally declining, as the model of Angel suggests?

In several European urban areas, the residential area is increasing at a much faster rate than its population. This trend towards reduced population densities, which started back in the early 1970s, is characteristic for small and medium-sized as well as large urban areas. Sprawl and the spread of low-density settlements are costly to provide with public transportation, services and other supplies, leading to an increasing reliance on private cars air pollution, and an overexploit of natural resources [81,110]. There is no sign that this trend is slowing down and, as a result, the demand for land around urban areas is remaining a crucial issue [54,58]. Our results are therefore in line with Angel et al. [6], because more cities experienced expansive growth with increasing rates after the year 2000.

However, dedensification and growth is by no way the universal trend for today’s urban areas. In contrast to Angel et al. [7], our findings stress the increasing role that shrinking urban areas are playing in dedensification processes (S3 Fig). This mainly results from a considerable population decline over just a few years, particularly in post-socialist Eastern Europe and in post-industrial regions in Western Europe. Especially after 2000, rates of density decreases in shrinking urban areas reached values comparable to those of growing ones. Because the number of shrinking urban areas increased until 2010, as a consequence of negative birth rates and negative net-migration, it can be expected that population decline with ageing will probably continue all over the continent in the next decades. This is most probably accompanied by a disproportional development of physical space as population loss impacts infrastructure usage, economies, productivity, and investment decisions, which, in turn, have impacts on land use [84,111].

Extensive construction activities parallel to population decline lead to an increasing disparity between a declining housing demand and an increasing supply of residential housing [9]. The seeming discrepancy between lacking demand and large-scale investment due to subsidies has received insufficient attention in contemporary urban research [112] and has not yet been included as an urban development path by the model introduced by Angel [6,7]. Thus, sprawl under population shrinkage conditions has implications for density changes similar to those that we find in growing, sprawling urban areas. Scholars and planners can benefit from paying more attention to the strong environmental losses of expansive soil-sealing that threatens biodiversity and increases the risk of both flooding and water scarcity [84,110].

### To what extent do we find differences in density changes between growing and shrinking as well as between small and large urban areas?

Densities significantly decreased in growing, sprawling and shrinking urban areas after the year 2000 (S3 Fig). In both contexts, an increase in residential area does not reflect population dynamics but is predominantly driven by the expansion of residential area. Moreover, the number of compact, growing urban areas with a stronger population influx and rapidly increasing densities decreased after 2000. Instead, we detect two increasingly important phenomena for densification processes in Europe that emerged after the turn of the millennium.

The first phenomenon characterizes urban areas with increasing densities, due to a reduction of residential areas by demolition or moratoriums on housing construction in suburban areas, in order to strengthen inward developments [107,109]. For declining urban areas, this refers to extensive demolition activities. This compact shrinkage is an interesting case of urban development because it provides an opportunity for densification and compaction based on slowed or halted population decline and a respective reduced housing demand which fosters inward growth and might lead to a smaller built-up area at the end as planning literature suggests [110].

The second densification phenomenon characterizes urban areas with a substantial population increase but without a corresponding increase in the residential area. This refers to continuously growing urban areas, such as those in Munich or Bilbao, but particularly to urban areas that experienced a considerable population loss but were able to ‘benefit’ from the refurbishment and reuse of existing vacant building structures, providing clear evidence of reurbanisation and regrowth of cities in Europe such as in Leipzig [9,20,113,114].

Both processes—the demolition of buildings in declining urban areas and renewed growth driven by population gain—are of increasing importance, fostering densification processes in Europe. Thus, these types of dynamics would have to be added to the types introduced by Angel [6,7,70], whereby the regrowth process, in particular, contributes substantially to a densification of urban land area (S3 Fig). Moreover, we identify an increasing polarization between growth and shrinkage when it comes to city size. Larger urban areas benefit more from population influx without the need for further expansion of residential areas, so far (reurbanisation), whereas smaller ones may grow at the expense of their hinterland, leading to decline in the near or more remote future. Although we basically agree with Angel et al. [7] that density change rates are lower for larger European urban areas (>100,000 inhabitants) than for small and medium-sized ones, we emphasize that regrowing urban areas represent an exception here: Their rates of density increase exceed those of other types, basically because the residential area does hardly expand that fast.

### Which driving factors might explain the observed trends?

Density change rates for European urban areas are diverging for what different factors come into play. Factors such as growth in GDP, purchasing power and household income are only able to explain land consumption to a certain extent [55,71]. Moreover, we would like to discuss three factors which share high explanation power for our analysis; their empirical investigation is, however, beyond the scope of this paper and thus requires further research.

#### Planning and institutional factors

A major reason for the variety of density changes lies in planning differences, in particular in national planning systems, the size of local governments and institutional fragmentation [55,115]. Changing spatial planning systems and a lack of trust in planning regulations has led to extensive sprawl in post-socialist Europe (the Baltic States, Ex-Yugoslavia, Ex-Czechoslovakia, Poland) from the early 1990s onwards [116,117]. As a consequence, massive construction activities with few constraints on land use or in the absence of master plans are observed in growing but especially in shrinking urban areas, which is especially pronounced in Romania and Bulgaria [118,119]. At the same time, national planning authorities hardly estimate the impact which a reinforced population loss might have on their urban systems [117]. Consequently, sprawl of growing urban areas is observed also in larger (capital) urban areas in Eastern Europe (Poland, Hungary) but also in Southern (Spain, Greece, Italy) and Western Europe (Ireland, France) confirming previous studies [5,54,55]. However, the East German example (e.g., Leipzig, [9,11]) shows that the increase in construction activities in shrinking urban areas is also part of a strategy targeted at stabilizing the housing market, especially because of a neglected building stock. For many countries the variety of responsible administrative actors at different levels is an additional barrier for comprehensive planning. However, several countries follow integrated planning approaches from the national down to the local level focusing on compact inner-city development. This goes along with a strong role of local planning such as in Germany, Scandinavia, Switzerland or the UK, leaving urban sprawl much less attractive behind and lead to a high percentage of compact urban growth [19,80].

#### Housing market mechanisms

The way supply and demand is balanced on the housing market is essential influencing density changes of cities. First, the share of rented vs owned flats impacts the residential choice of households and can pull certain household groups e.g. young and single households into rented apartments with the flexibility to move again within the housing market. Second, this is determined by the actor constellation as in the European context larger cooperatives, partly owned by municipalities, hold a high share of flats and essentially determine to what extend an extension of residential area is necessary or obsolete. Third, the privatization of the housing stock together with cheap and available land [17] has led to extensive construction activities more sparsely, as free-standing and hence more space-consuming building structures [20,80,120] in particular in Eastern and Southern Europe, also driven by the growth of coastal tourism in Spain or France [59]. Planning need to counteract this dedensification trend by, for example, repurchasing private land and prevent it from further speculative housing stock [107].

#### Investments and economic interventions

In some regions, residential area was adapted to population loss which in turn resulted in different expressions of density changes. Thereby, national (transfer) programs particularly pronounced in welfare states, for example, in Germany or in the UK, had considerable impact on this urban development [9,10]. The large restoration state programme in eastern Germany, “Stadtumbau Ost”, aimed at removing housing surplus as well as adapting social and technical infrastructure facilities (e.g. schools, kindergardens, transportation, energy and water supply). This is an obvious difference e.g. to US-cities which are largely influenced by private investments for construction and refurbishment. These public investments stabilized the housing market has led to the phenomenon of regrowth and an associated population growth without land expansion [9]. The stabilization of population numbers and reuse and revitalization usually start in selected inner parts of cities, which offer better infrastructures, cultural and educational facilities, as well as green and recreational spaces [10,11,107,109]. Finally, the German example shows that urban sprawl significantly slowed down with the phasing out of state-initiated tax policy supporting single-family houses in 2006.

## 5. Conclusions

Our study showed that dedensification did not slow down but, instead, intensified after the turn of the millennium and has therefore continued to dominate urban development in Europe [6,54,55,121]. However, in comparison to the concept introduced by Angel et al. [6], we can draw conclusions about three basic differences, or the fine-tuning that is required with respect to our findings:

1. We need a more differentiated view on the continental scale: All data values analysed are very well spread, compared to the diagram presented by Angel et al. [6], which shows the overall difference between the European sample and the global sample that the authors used. Apart from urban sprawl, the patterns identified by Angel et al. [6] are more similar to those patterns that shrinking urban areas with decreasing densities show, in our European sample. It is worth asking about the extent to which urban shrinkage plays a role in the concept established by Angel et al. [6] and about the extent to which the patterns of their global sample are a result of a slowing in population growth. A reinforced population loss, an adapted residential area within urban areas as well as an increasing polarization between larger and smaller cities in Europe, which is largely dominated by smaller settlements, are three trends which result in interrelation to the various drivers mentioned above to different expressions of density changes that might be specific for Europe, e.g. compared to US cities. Although Europe shows tremendous regional variations such as between (post-socialist) Eastern and Southern Europe and can thus not be regarded as having a uniform continental trend, a continental perspective following Angels’ ideas, would help to uncover the multiple dynamics that have been described.
2. There is a need to enlarge the perspective: It should be emphasized that population dynamics fluctuate much more than a change in the physical shape of an urban area. On the one hand, housing and infrastructure investments tend to have a long life-span and show a considerable inertness with slower rates of change. On the other hand, international and local migration patterns may change very quickly, leading to different density changes, depending on the size and the location of the urban area. The sample used in this study encompasses the entire range of city size and demonstrates that certain processes, such as sprawl, are characteristic for small and medium-sized as well as large urban areas, whereas large urban areas currently experience reconcentration processes, without any additional physical expansion. This reconcentration, which occurs parallel to (ongoing) sprawl, was already observed, in the 1980s, in Northern and Western Europe (measured by population development [121]). However, residential densities that are too high might imply disadvantages such as price increases for housing, rising infrastructure costs, pollution, and additional costs of a degraded environment and related health problems [59]. Both aspects indicate a polarization between large and small urban areas at the continental scale and are more likely to continue in the future. Therefore, a more detailed discussion of the consequences of this polarization together with the interdependencies of cities is required; this is certainly an issue for planning.
3. The analysis concept used in this paper has shown the advantage of using both indicators (the population and the built-up area) in order to reveal different patterns of density changes. This enriches the model by Angel et al. [7] by looking at both shrinking and growing urban areas and demonstrates trends that could remain hidden from view at a larger (e.g., global) scale of analysis. This helps to better understand why population growth alone is not sufficient to explain the growth in urban land consumption [17,54,116]. However, the explanatory power of our study is limited by the data used and further empirical research is needed in order to fully uncover the drivers mentioned above. As various local indicators such as GDP is hardly available nor comparable the quantitative detection of density patterns presented in this paper can be combined by national to local focused studies. First, a local governance analysis covering one up to several case studies can be performed within a regional or national planning context [115] in order to investigate the of planning for the extension or reduction of housing supply together with an analysis of compactness, adaptation and amenity measurements of local authorities. Second, an analysis of economic development should be necessarily accompanied by an investigation of housing supply and demand. This involves different residential migration groups as well as price-ownership constellation and public to private investments into housing and infrastructure. We strongly recommend for both future research orientations to spatially focus the study to regions or nations and necessarily include the urban hinterland into the observation as cities are not isolated entities [122].

Based on the typology presented here, it can be expected that existing structures are impacting on future evolution, continuously driven by land prices, as well as people’s preferences and lifestyle. Thus, the approach provides some anchor points for a more context discussion backed up by multiple data, which is desirable for policy and planning aiming at compact structures. It should therefore be seen as a starting point and an initial endeavour that pleads for a differentiated and contextualized view on urban density changes and its explanatory power in complex settings.

## Supporting information

S1 Fig Comparison of delineation of urban areas—Morphological Urban Area (MUA), Urban Morphological Zone (UMZ) and Urban Area (UA)–used in the paper. (TIF)

S2 Fig Comparison of median values of population size and surface of residential area (urban fabric) for countries (authors’ calculations). (TIF)

S3 Fig Six different types of density changes for growing and declining urban areas in different periods. (TIF)

S1 Table Basic statistics for countries. (XLSX)

S2 Table Basic statistics on changes [Δ] of population, residential area, and density. (XLSX)
