# Supplementary figures and images for: Compact or spread? A quantitative spatial model of urban areas in Europe since 1990
Source: PLoS One. 2018 Feb 28;13(2):e0192326. doi: 10.1371/journal.pone.0192326 (PMC5830312; doi:10.1371/journal.pone.0192326)

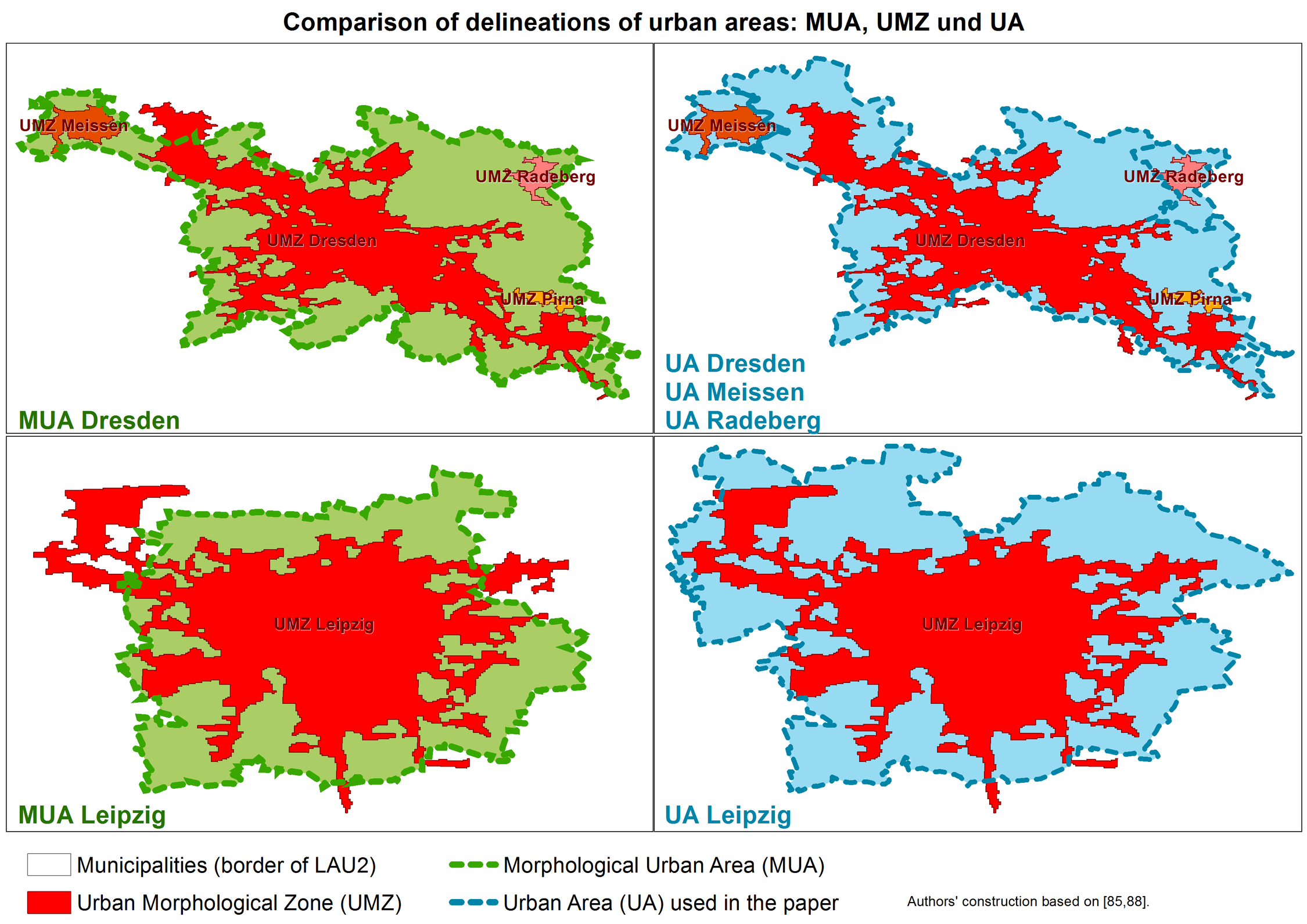

Supplement: S1 Fig — (TIF) [file pone.0192326.s001.tif]

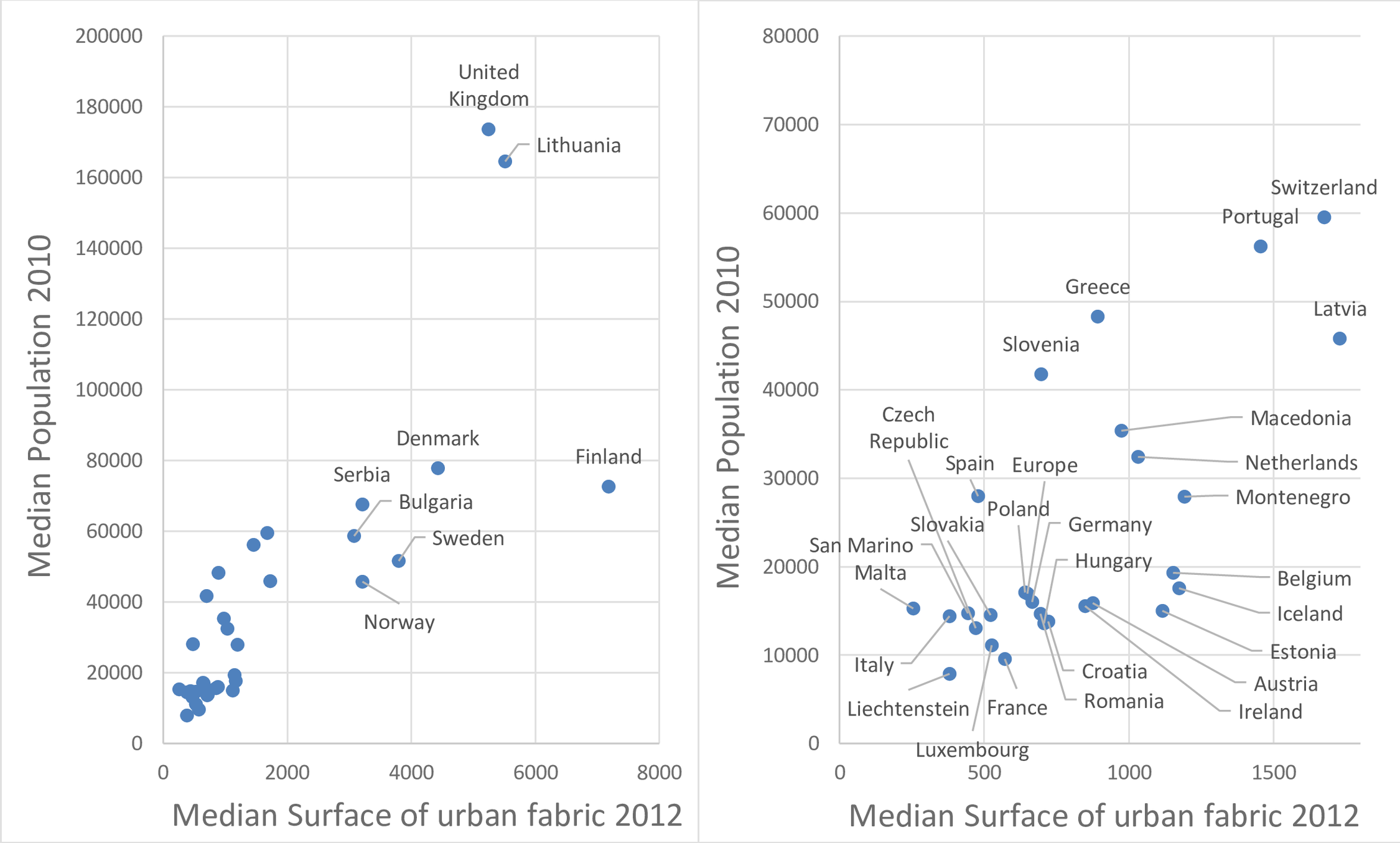

Supplement: S2 Fig — (TIF) [file pone.0192326.s002.tif]

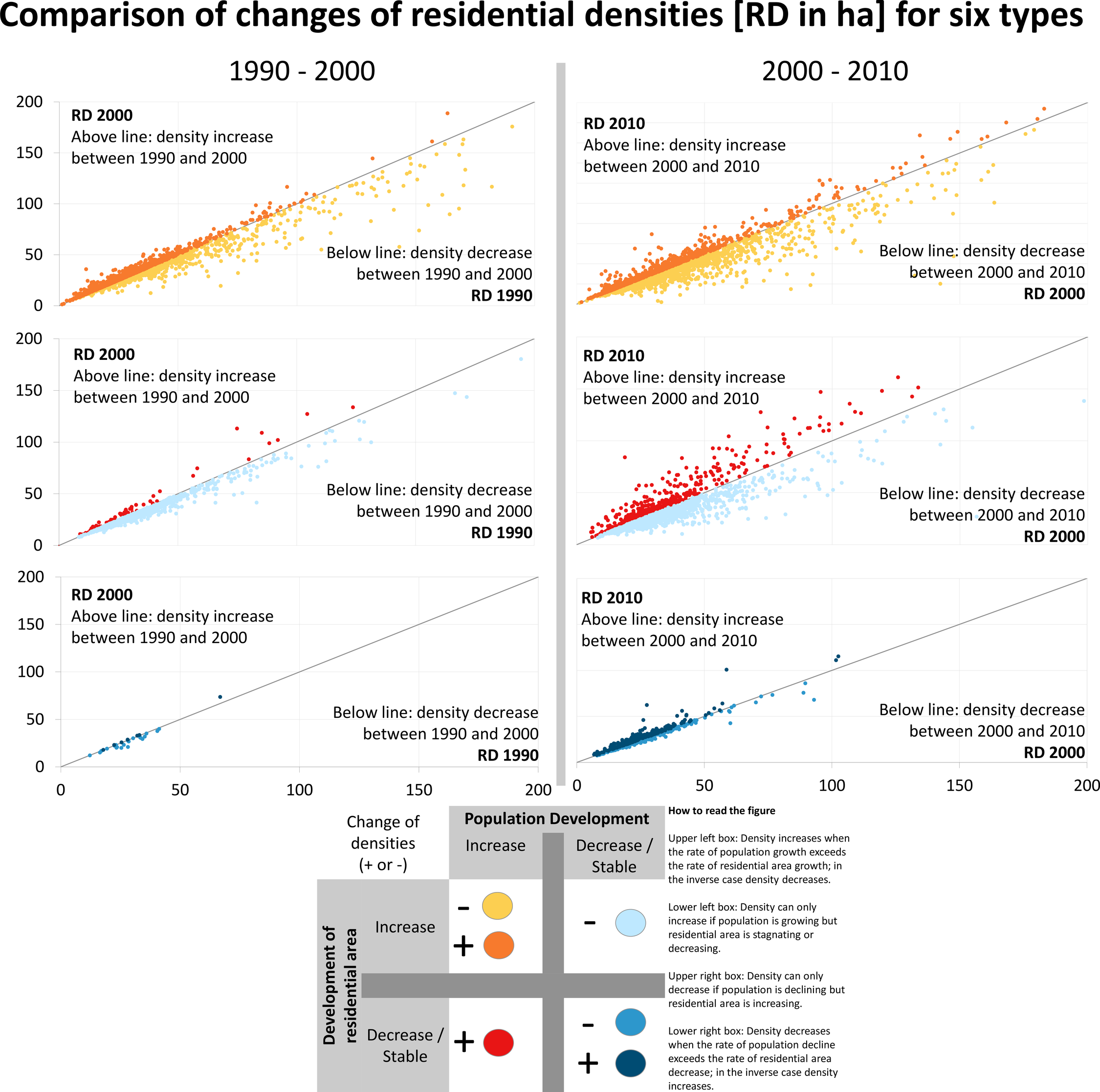

Supplement: S3 Fig — (TIF) [file pone.0192326.s003.tif]
